# New View on Dietary Fiber Selection for Predictable Shifts in Gut Microbiota

**DOI:** 10.1128/mBio.02179-19

**Published:** 2020-02-18

**Authors:** T. M. Cantu-Jungles, B. R. Hamaker

**Affiliations:** a Department of Food Science, Whistler Center for Carbohydrate Research, Purdue University, West Lafayette, Indiana, USA; Corporación CorpoGen Research Institute

**Keywords:** dietary fiber, gut microbiota, fiber specificity, fiber response

## Abstract

Dietary fibers can be utilized to shape the human gut microbiota. However, the outcomes from most dietary fibers currently used as prebiotics are a result of competition between microbes with overlapping abilities to utilize these fibers. Thus, divergent fiber responses are observed across individuals harboring distinct microbial communities. Here, we propose that dietary fibers can be classified hierarchically according to their specificity toward gut microbes. Highly specific fibers harbor chemical and physical characteristics that allow them to be utilized by only a narrow group of bacteria within the gut, reducing competition for that substrate. The use of such fibers as prebiotics targeted to specific microbes would result in predictable shifts independent of the background microbial composition.

## Abstract (precis)

Dietary fibers can be utilized to shape the human gut microbiota. However, the outcomes from most dietary fibers currently used as prebiotics are a result of competition between microbes with overlapping abilities to utilize these fibers. Thus, divergent fiber responses are observed across individuals harboring distinct microbial communities. Here, we propose that dietary fibers can be classified hierarchically according to their specificity toward gut microbes. Highly specific fibers harbor chemical and physical characteristics that allow them to be utilized by only a narrow group of bacteria within the gut, reducing competition for that substrate.

## PERSPECTIVE

The human colon harbors a dynamic and complex community of microbes and holds one of the highest cell densities known (1). Microbiota-host interactions not only impact the host digestive tract but are also involved in many immunological and physiological responses that affect distinct body sites and systems (2). Not surprisingly, many gut bacterial species/groups and their produced metabolites have been related to the course, prevention, or treatment of diseases such as diabetes, obesity, hyperoxaluria, ulcerative colitis, and cancer (3). Thus, the manipulation of commensal gut bacteria is a potential strategy in the management of several health conditions (4). Diet has a pivotal role influencing the composition and function of intestinal microbes and, in this sense, has been recently explored as a tool to shape the gut microbiota (4, 5). A typical diet in Western countries supplies the colonic microbes with 13 to 20 g of dietary fiber (DF) daily (6–8) on which commensal bacteria primarily rely to harvest energy and carbon. As carbohydrate polymers and oligomers, DFs are composed of a variety of monosaccharides polymerized through distinct linkage patterns to final molecules of diverse size ranges. They may be further linked to other chemical groups or molecules (e.g., acetyl, methyl, and feruloyl groups) and possess physical variations, such as solubility degree, viscosity, and three-dimensional arrangements. The vast array of simple and complex possible structures means that DFs contain challenging substrates that require sophisticated bacterial machineries to be accessed, degraded, and utilized (9). Microbes possess genetic information to express specific carbohydrate-active enzymes (CAZymes), recognition and binding proteins, and transporters that are required in this process (10). The heterologous expression of this molecular machinery in distinct species results in divergent specialization to ferment discrete fiber structures (9). Conceivably, a dietary fiber structural alignment to specific bacterial abilities would allow selective stimulation of growth and/or activity of microbes associated with health protection and well-being.

## CHALLENGES FOR A TARGET-SPECIFIC FIBER APPROACH

Although microbes have specific abilities to utilize distinct DF structures, it seems not so easy to simply give a particular DF to a person to promote a specific gut bacterium. For instance, commonly used prebiotic fibers do not present consistent results across individuals. We believe that this is in part because there are easily accessible and simple fiber structures in nature that are utilized by many bacteria (low-specificity fibers). For example, fructooligosaccharides (FOS), a soluble short-chain DF polymer containing mainly β(1→2)-linked fructose that is commonly used in clinical trials to promote shifts in the gut microbiota, was initially associated with the growth of bifidobacteria (11). More recently however, Scott et al. found that of 14 distinct bacterial species tested, including representative members of the *Bacteroidetes*, *Firmicutes*, and *Actinobacteria* phyla, all were able to grow well on FOS using *in vitro* single-culture experiments (12). An *in vivo* study using 16S rRNA gene sequencing analysis further showed that many other bacteria (102 taxa) were stimulated or inhibited to some extent by FOS supplementation in mice (13).

The coexistence of many different microbes able to utilize the same DF, accompanied by high cell densities relative to the available nutrient resources, results in competitive pressures within the gut that dictate different fiber response outcomes (14). In that way, the bacterial groups stimulated by a given low-specificity DF will differ when community pressures are different, i.e., in distinct microbial communities. Venkataraman et al. evaluated butyrate production during *in vitro* fecal fermentation of resistant starch, another DF that many bacteria have the ability to utilize (15). The results presented show varied responses in samples from different subjects, presumably due to distinct initial characteristics of their gut microbiota. Recently, Johnson et al. conducted a human study, without diet interventions, to investigate how dietary patterns relate to microbial shifts based on daily fecal sampling and dietary records (16). Although they found that diet significantly alters the gut microbiota, distinct personalized bacterial responses were observed among individuals consuming the same groups of food. This is likely due to the DF response being dependent on the composition of the microbial community. In a recent study with germ-free mice fed a diet containing arabinoxylan and colonized with an artificial community, including the arabinoxylan degraders Bacteroides ovatus and Bacteroides cellulosilyticus, *B. ovatus* increased only when *B. cellulosilyticus* was absent, showing that fiber response is closely related to microbial community layout (17). Chen et al. showed that fecal ferments with an initial dominance of Prevotella spp. versus Bacteroides spp. respond differently to DFs regarding both bacterial shifts and metabolites produced during *in vitro* fermentation (18). Notably, classifying the human microbiota only by enterotype (*Prevotella* spp. versus *Bacteroides* spp.) is a generalist approach (19), and it is probable that distinct fiber responses happen within individuals classified in the same enterotype due to divergences in microbial communities. Data from the Human Microbiome Project (20) show that although individuals have up to several hundred species of microbes within their gut, thousands or more different species inhabit the gut of human populations collectively, which confirms a high degree of variation in microbiota composition among individuals. From a functional point of view, such high variability would also infer a range of divergent (and perhaps unpredictable) responses when a low-specificity fiber is given to different individuals (18, 20). Thus, the use of these fibers to sustain the growth of targeted bacteria in a predictable way in every individual hardly seems an achievable goal.

On the other hand, fibers with higher specificity (i.e., accessible and fermentable by a limited number of bacteria) could promote specific taxa independent of the competitive pressures of the environment for nutrient acquisition. As an example, Shepherd et al. colonized three groups of mice harboring distinct microbiota communities with a rare Bacteroides ovatus strain isolated specifically for its ability to utilize both inulin and the polysaccharide porphyran (21). Because no other bacteria in mouse intestinal communities could utilize porphyran, its administration led to a targeted, predictable, and dose-dependent increase in this rare *B. ovatus* population. Moreover, different *B. ovatus* growth responses were observed among the three mice microbiotas when inulin was used as the only energy source; however, when porphyran was utilized, the growth rate was consistent independent of the background microbiota (21). We have also shown that specific and unusual dietary fibers can be utilized to target the bacterial growth of species of biological significance that naturally occur in the gut. Using a fungal insoluble β-(1-3)-linked glucan, a genus of butyrate-producing bacteria (Anaerostipes) was specifically stimulated in an *in vitro* human fecal fermentation, increasing abundance in 24 h from <0.5% of the total bacteria in the initial inoculum to approximately 24% (22). It seems that this fiber is highly specific for these bacteria, with not many other microbes in the gut having the ability to compete and utilize these β-glucans, although an ecological effect cannot be dismissed and is under investigation. Also, entrapment of starch into alginate microspheres was shown to reduce starch utilization by *Bacteroidetes* species and specifically promoted butyrogenic *Firmicutes* (23). While the starch utilization system (Sus) in *Bacteroidetes* requires physical attachment to degrade and utilize starch (24), *Firmicutes* employ cellulosome-like appendages or secrete starch-degrading enzymes with no need for direct physical attachment to the fiber, taking advantage of the inaccessible alginate-entrapped starch for growth.

## HIERARCHICAL DIETARY FIBER MODEL FOR MICROBIAL SPECIFICITY

**FIG 1:**
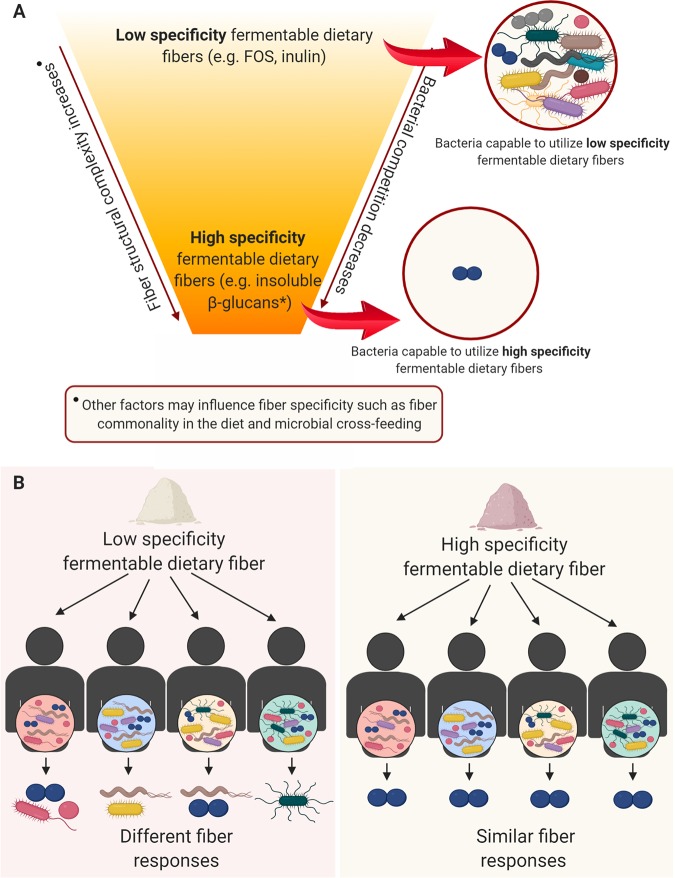
(A) A hierarchical view of dietary fiber specificity toward gut microbes. *, Cantu-Jungles et al. (22). (B) Fiber responses among individuals using low- versus high-specificity dietary fibers.

We propose that dietary fibers can be generally classified hierarchically according to their specificity to gut microbes (Fig. 1A). On the top of the hierarchy are low-specificity fibers that are easily accessible and utilized by many colonic microbes, resulting in competitive pressures to utilize these nutrients (Fig. 1A) and variance in the response related to an individual’s gut microbiota community structure (Fig. 1B). One could classify FOS and inulin as low-specificity DFs because many bacterial taxa are able to access and degrade them. The fermentation response to these DFs largely rely on microbes’ ability to compete among each other to utilize them, with competitive pressures varying among individuals as much as microbial community composition differs. Thus, the use of low-specificity dietary fibers would generate divergent fiber responses across individuals. On the other hand, at the bottom of the hierarchy are high-specificity fibers, such as the above-mentioned insoluble β-glucans, that possess structural features that only few bacteria can access, degrade, and utilize efficiently. These include DFs with both complex chemical (sugar compositions and linkage combinations) and physical (e.g., insoluble matrix fibers) structures. Due to the more specific alignment of these DF structures and lower number of utilizing bacteria, competition for these highly specific fibers is reduced. With a limited number of microbes able to access and degrade them, high-specificity DFs promote a more targeted action toward their utilizers (Fig. 1A). Distinct intermediate levels of fiber specificity may take place according to physicochemical structures that would confer the fibers with a higher or lower degree of specificity. We believe that the reduced competitiveness for high-specificity fibers allows a more predictable and similar fiber response in a population, even in individuals harboring distinctly different microbial communities but that contain the target bacteria (Fig. 1B). Importantly, the targeted bacteria to be promoted by highly specific fibers should be either naturally present in one’s microbiota, as shown by Cantu-Jungles et al. (22), or supplemented as a probiotic with the addition of the highly specific fiber as shown by Shepherd et al. (21). We acknowledge that a given target bacterium may not be prevalent in a population, but still, the high-specificity fiber approach would be valid as a prebiotic if the bacterium is present or synbiotic if absent.

Many other fermentable DFs, such as arabinogalactans, mannans, xylans, arabinoxylans, xyloglucans, pectins, resistant starch, soluble glucans, and arabinans, exist in nature (25). Also, distinct fiber characteristics (as discussed in Dietary Fiber Characteristics That May Affect Specificity) within the same class of dietary fiber would confer a higher or lower specificity toward a targeted gut microbe. A comprehensive classification of all distinct DFs regarding specificity to gut bacteria is yet a matter of investigation but could conceivably result in a compilation of a library of DF structures to support a range of beneficial gut bacteria (9). This would necessitate a further understanding of key bacterial species that are important to gut health and then a determination of the DF types and structures that support them. Moreover, cross-feeding occurs within gut commensals and further increases the number of species that benefit from the presence of a given DF (26). Yet, there is also evidence that bacteria are not strictly assigned as cross-feeders, and when other carbohydrate sources are available, they can directly utilize them (27). Nonetheless, discrete fiber characteristics, including chemical and physical structures, are important features to DF specificity and could be selected or manipulated to increase a DF’s specificity to a particular bacterium or bacterial group.

## DIETARY FIBER CHARACTERISTICS THAT MAY AFFECT SPECIFICITY

### Physicochemical complexity.

The molecular machinery necessary to ferment a fiber is structure specific; hence, DF chemical and physical structures largely influence which bacteria will access and ferment them (9, 28, 29). More-complex DF chemical structures (e.g., those containing a variety of sugars, linkage types, and branching patterns) require many bacterial enzymes to act in synergy for their complete saccharification, and, from a rational point of view, there is a tendency that the more complex the DF, the fewer the bacteria in the gut capable of fermenting it. For instance, while many *Bacteroides* species can grow on xylose and glucose, only a limited number of taxa harbor the genetic machinery to grow in xyloglucans (30, 31). Recently, Ndeh et al. explored the ability of 29 *Bacteroidetes* strains to grow on type II rhamnogalacturonan, a pectic polymer containing 21 distinct glycosidic linkages (32). Less than one-third of these organisms grew on the glycan. Scott et al. have shown that polymerization degree also influences fiber specificity. In single bacterial cultures, all 15 evaluated taxa (10 representative *Firmicutes*, 3 Bifidobacterium spp., and 2 Bacteroides spp.) could grow on short-chain FOS, but only five of them grew on a long-chain inulin (12). Overall, these data indicate that more-complex fiber structures are likely to be more selective to specific bacteria than are simple DF polymers.

While there are reports of human supplement studies of specific responses to more simple dietary fibers, upon close inspection of the results, one sees varied responses ranging from increases to decreases in both metabolites and target bacteria. For instance, galactooligosaccharides were shown to specifically increase Bifidobacterium spp.; however, only 11 out of the 18 subjects tested had actual increases in *Bifidobacterium* spp. (33). Also, Vandeputte et al. (34) reported specific responses to inulin-based fructans, but there was high variability around the mean for *Bifidobacterium* spp., suggesting responders and nonresponders. Baxter et al. (35) showed that in 43 individuals supplemented with resistant starch, only 22 individuals responded with a butyrate increase, while 21 individuals responded with a reduction or no changes in fecal butyrate concentration. Bacterial shifts, including those of Ruminococcus bromii, a known starch degrader, also showed high variability among subjects.

**FIG 2:**
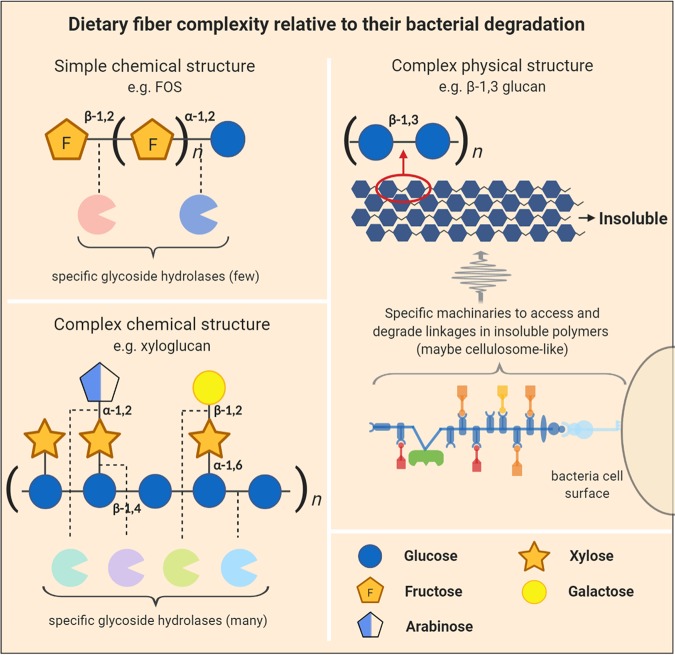
Dietary fiber complexity relative to their bacterial degradation. Dietary fibers with simple chemical structures, such as fructooligosaccharides (FOS), require few bacterial glycoside hydrolases to degrade them, whereas more-complex molecules, such as xyloglucans, which contain a range of sugar and linkage types, require that bacteria have more glycoside hydrolases for their complete degradation. Complex physical structures, such as those found in insoluble dietary fibers (e.g., β-1,3 glucan [22]), also require that bacteria have specific and perhaps more complex machinery to access these insoluble substrates (maybe cellulosome-like appendages).

Another important point regarding fiber specificity to bacteria involves physical properties, such as insolubility degree, that reduce the accessibility of DFs by microbes and provide an additional challenge for attachment and enzymatic degradation. Leitch et al. demonstrated that specialized groups from the *Firmicutes* phyla, such as Clostridium clusters *IV* and *XIVa*, are more associated with insoluble particles in human feces than are *Bacteroidetes* (36). Differences in bacterial motility may be particularly important in colonizing this kind of substrate. For instance, Roseburia inulinivorans, a bacterium from the *Clostridium* cluster *XIVa*, had genes related to flagellar synthesis that are upregulated during growth on starch (insoluble fiber) but not on inulin (soluble fiber) (37). Moreover, bacteria possessing cellulosome-like appendages, which allow bacteria to access insoluble substrate matrices, also have an advantage in the utilization of these polymers (38). Thus, DF specificity to bacteria with these kinds of apparatuses could be increased by the utilization of its insoluble forms. Physical accessibility can also be manipulated to increase specificity to a target group of microbes. We have recently shown that a solubilized corn arabinoxylan that was cross-linked to form soluble matrices shifted growth toward butyrogenic *Clostridia* bacteria (39). Also, as mentioned above, raw starch entrapped in porous alginate microspheres was shown to promote butyrogenic *Firmicutes* in mice, with a reduction in *Bacteroidetes* species that must physically attach to normal resistant starch by the Sus assembly to utilize it (23). *Firmicutes* harbor distinct starch utilization strategies that do not require physical attachment. There is some evidence that other physical characteristics are relevant during DF fermentation as well. In mice, divergent bacterial populations in the cecum were promoted by the same diet in different physical forms (powdered versus pelleted) (40). Also, increases in viscosity were related to the growth of total anaerobes and *Clostridium* spp. in a gastrointestinal simulator inoculated with fecal microbiota, and the decrease in viscosity was related to Enterococcus sp. growth (41). Recently, Tuncil et al. showed that in *in vitro* fecal ferments, larger wheat bran particles selected toward a more butyrogenic microbiota, while smaller particles were associated with a more propiogenic microbiota (42). Examples of how fiber structural complexity is addressed by bacteria are illustrated in Fig. 2.

### Other factors that may affect fiber specificity.

Besides dietary fiber physicostructural complexity, we believe that some other factors may influence the degree of fiber specificity, such as DF commonality in diets and utilization through bacterial cross-feeding.

From an evolutionary point of view, it is plausible to think that fewer bacteria in the human gut are equipped to digest DFs that are rarely consumed in the diet. Bacterial genes not often utilized confer a fitness cost to the bacterium, which might drive to an adaptive process to get rid of these superfluous genes (43), such as those related to the digestion of DFs uncommonly consumed in the human diet. In fact, observations from a synthetic microbial community from the human gut show that the ability to grow in rarely consumed DFs, such as laminarin (from algae) and lichenin (from lichens), is restricted to few bacteria (44). As previously discussed, in our research group, a linear insoluble β-1,3 glucan resulted in the growth of specific bacteria in a human fecal gut community (22). Besides the complex physical structure (i.e., insoluble form) of the glucan, β-1,3 linkages between glucose units are mainly found in fungi, oomycetes, and lichens (45) and are not often consumed in large amounts in the human diet, therefore increasing its specificity to certain microbes. Accordingly, common DFs are likely to have multiple utilizing gut bacteria, while uncommon DFs would be utilized by few bacteria and therefore present more similar fiber responses among individuals who have the target bacteria.

Dietary fibers that support a limited number of bacteria would likely not be involved in cross-feeding, which involves degradation by a keystone species to release DF fragments or simple metabolic products that are used by other gut bacteria. A good example is resistant starch, which is degraded and utilized by a group of bacteria through cross-feeding, thus leading to less specificity (35). Thus, fibers that are more common in diets and are utilized through cross-feeding tend to have lower specificity, while fermentable fibers that are uncommon in the diet and those that do not result in cross-feeding tend to have higher specificity.

Overall, physicochemical fiber characteristics and these other factors combine to give DFs properties of low to high specificity regarding their utilization by gut bacteria.

## CONCLUSIONS

We believe that a niche differentiation of taxa with unique abilities to ferment highly specific DF structures naturally occurs in the gut. The design and selection of high-specificity DFs as the substrates for the modulation of the gut microbiota would prevent resource competition, resulting in more targeted and predictable shifts in specific taxa independent of the overall microbiota composition.
