# Erratum for Cantu-Jungles and Hamaker, “New View on Dietary Fiber Selection for Predictable Shifts in Gut Microbiota”

**DOI:** 10.1128/mBio.00747-20

**Published:** 2020-05-26

**Authors:** T. M. Cantu-Jungles, B. R. Hamaker

**Affiliations:** a Department of Food Science, Whistler Center for Carbohydrate Research, Purdue University, West Lafayette, Indiana, USA

## ERRATUM

Volume 11, no. 1, e02179-19, 2020, https://doi.org/10.1128/mBio.02179-19. The linkage type of fructooligosaccharides (FOS) in Fig. 2 and in the text (page 2 of the PDF version, paragraph 1) was incorrectly written as β-1,2 and should be β-2,1.

